# Supplementary material for: Cross-Talks between RKIP and YY1 through a Multilevel Bioinformatics Pan-Cancer Analysis
Source: Cancers (Basel). 2023 Oct 11;15(20):4932. doi: 10.3390/cancers15204932 (PMC10605344; doi:10.3390/cancers15204932)
Supplement: Supplementary file 1 [file cancers-15-04932-s001.zip › Table S1.pdf]

Immune cell abbreviation	Gene set signature of immune cell
CD4_naive	CD2/CD3G/CD4/CD40LG/GIMAP6/GLG1/HMOX2/IL7R/ITK/LIMD2/ LY9/NAA16/OBSCN/PACS1/PLCL1/RPL14/SEPT9/SNPH/TPP2/TRAF1/ZBTB40
CD8_naive	BLNK/CA14/CALY/CCDC87/CCR7/CD8A/CD8B/CRTAM/FXYD7/GJB4/ GPR15/HTR1B/KERA/KRT1/LIN28A/MAN1C1/MOGAT2/NKTR/PSG11/ RRH/SLC17A4/SMCP/SMR3B/TNKS2/TREM1/ZNF208
Cytotoxic	BLNK/CD8A/CD8B/GNLY/GZMA/GZMH/ITGAM/KLRK1/KNG1/PRF1/ PSORS1C2/PTGDR2/SCN3A/TNFRSF10C
Exhausted	ADGRG1/AFAP1L2/CCND2/CD38/CD8A/CD8B/CHST12/CTLA4/DFNB31/ EOMES/FUT8/ITM2A/LAG3/MYO1E/NDFIP2/PARK7/PDCD1/SIRPG/SNX9
Tr1	CCR4/CD28/CD4/LAX1/TNFRSF4
nTreg	CD4/CD5/CTLA4/DUSP4/FOXP3/IL10RA/IL2RA/SIT1/STAT5A/TNFRSF9
iTreg	ATG2B/CCR3/CCR4/CCR8/CD28/CD5/CTLA4/FASLG/FOXP3/GALNT8/ HS3ST3B1/ICOS/IL10RA/NFATC3/PPM1B/SIT1/STAT5A/TTN/ZFYVE9
Th1	APBB2/CCL4/CTLA4/EIF2B2/GGT1/IFNG/IL2/LTA/MNAT1/SLAMF1/ STAT1/SYNGR3/TACO1
Th2	GATA3/GSTA4/GZMK/IL4/SLC25A44
Th17	CD4/IL17RA/IL1R1/IL21/RORC

Table S1. The Table depicts the gene set signature of each immune cell used in ImmuCellAI analysis.
