# Supplementary figures and images for: Cross-Talks between RKIP and YY1 through a Multilevel Bioinformatics Pan-Cancer Analysis
Source: Cancers (Basel). 2023 Oct 11;15(20):4932. doi: 10.3390/cancers15204932 (PMC10605344; doi:10.3390/cancers15204932)

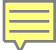

YY1

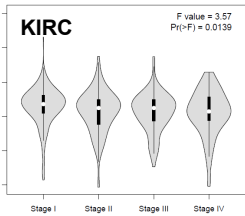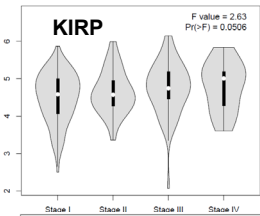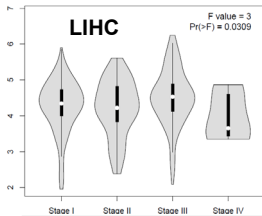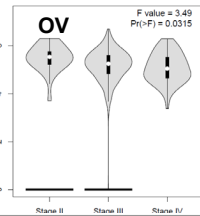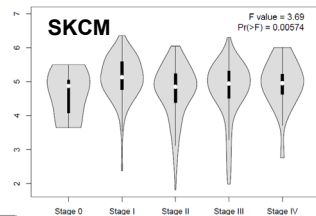

PEBP1

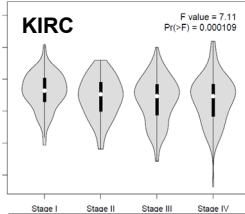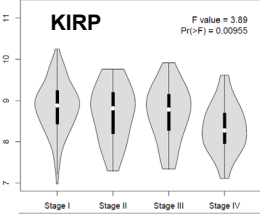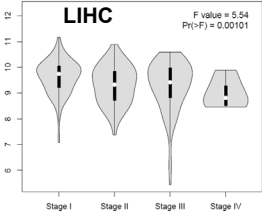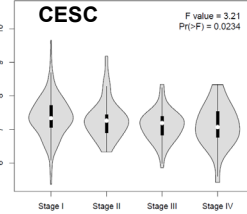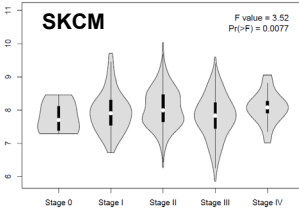

PEBP1

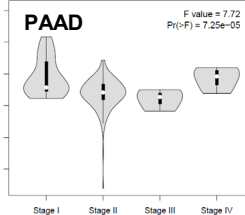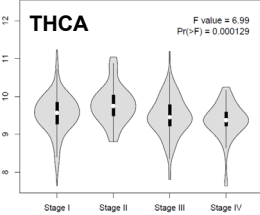

Supplement: Supplementary file 1 [file cancers-15-04932-s001.zip › Figure S1.pdf]

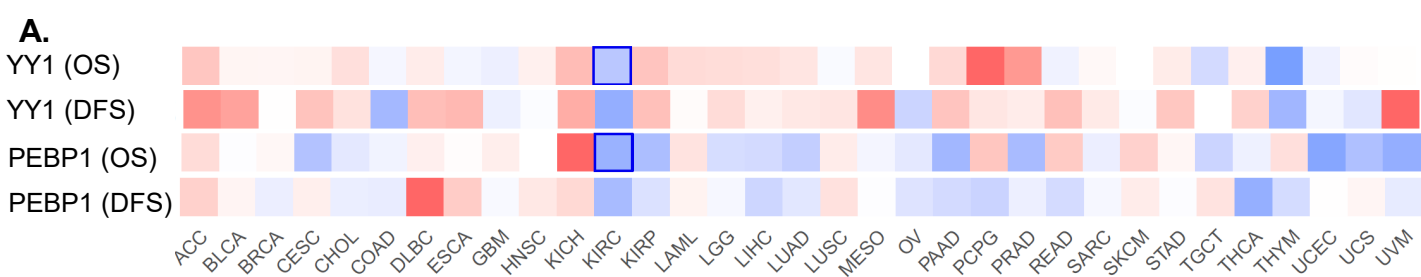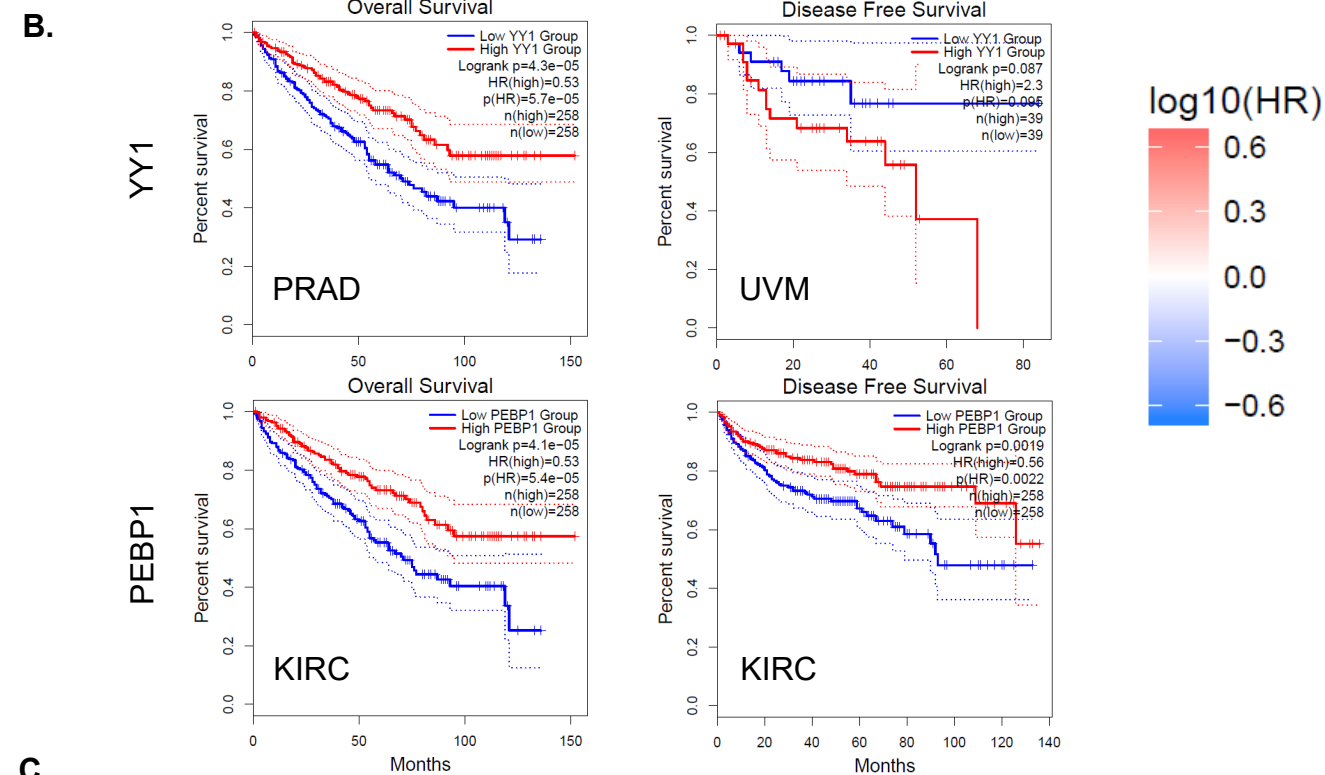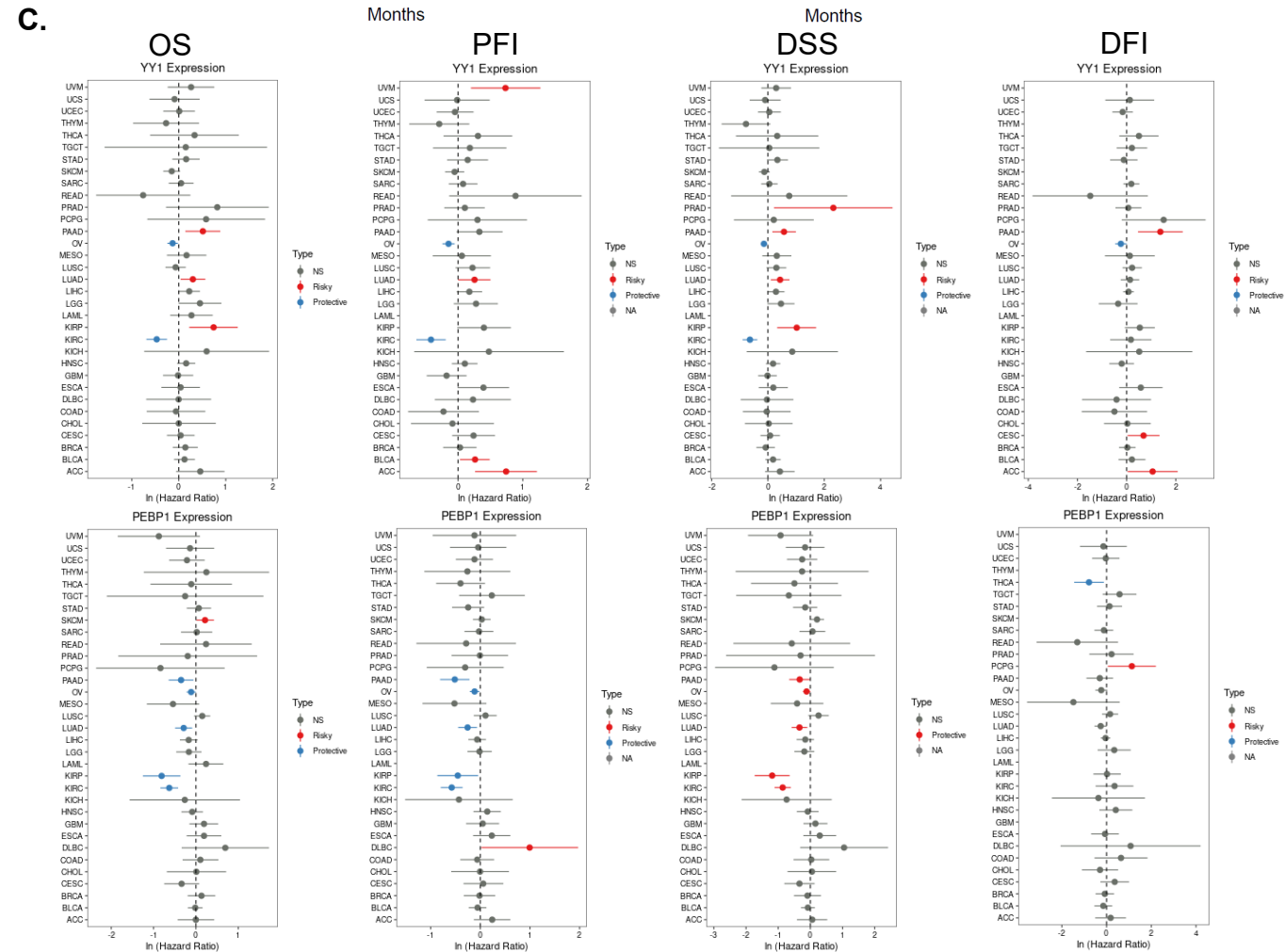

Supplement: Supplementary file 1 [file cancers-15-04932-s001.zip › Figure S2.pdf]

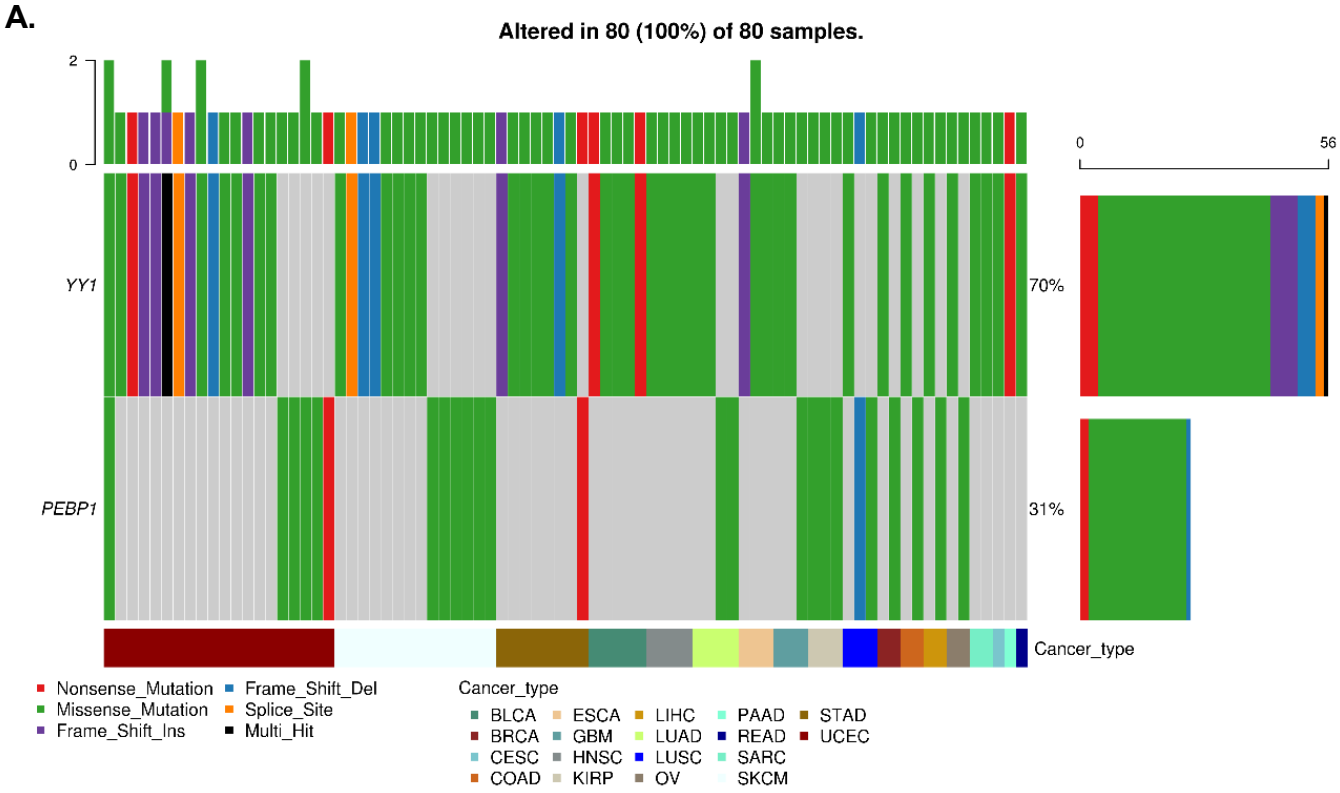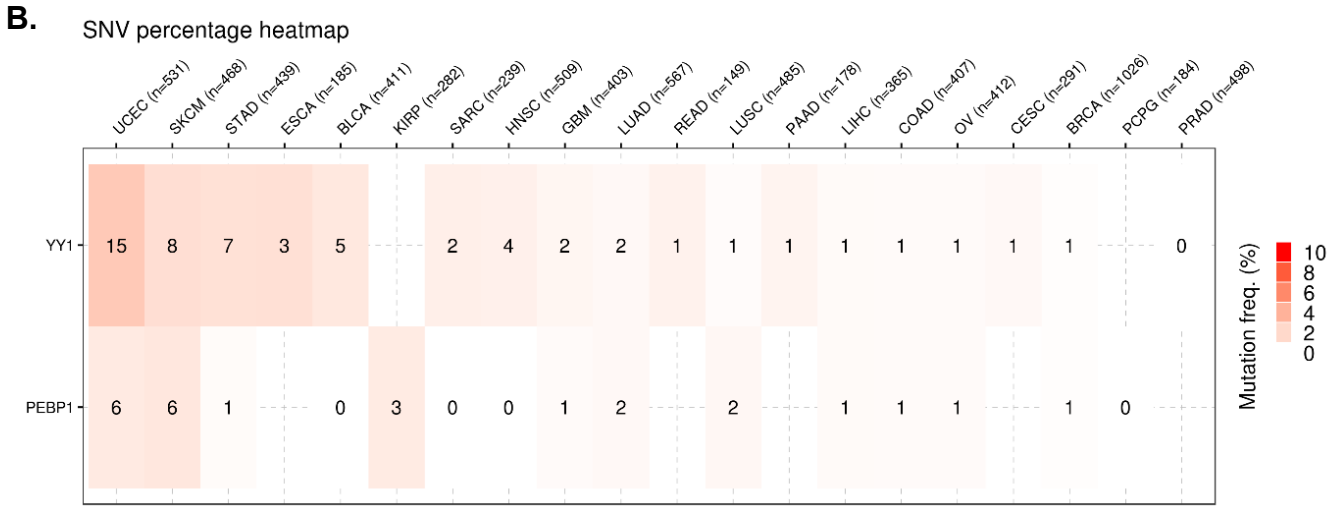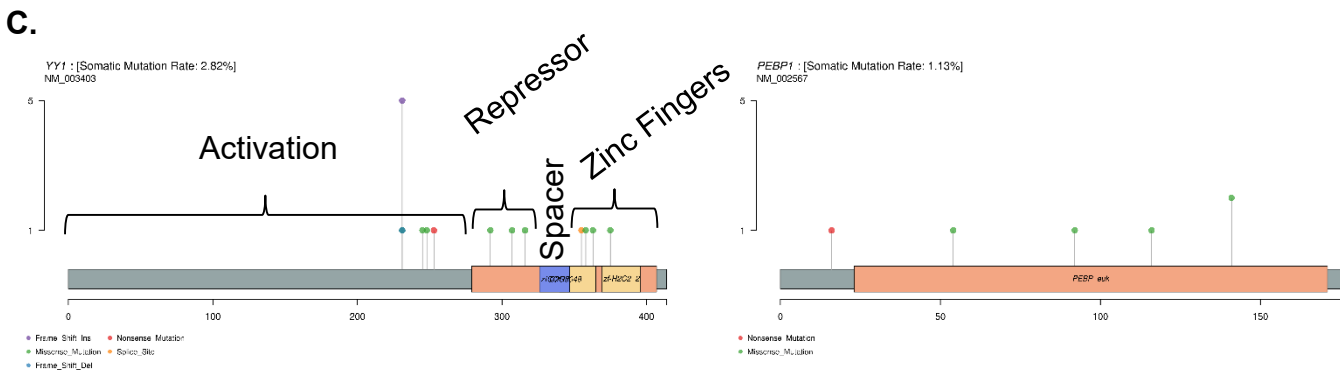

**Figure S3**

Supplement: Supplementary file 1 [file cancers-15-04932-s001.zip › Figure S3.pdf]

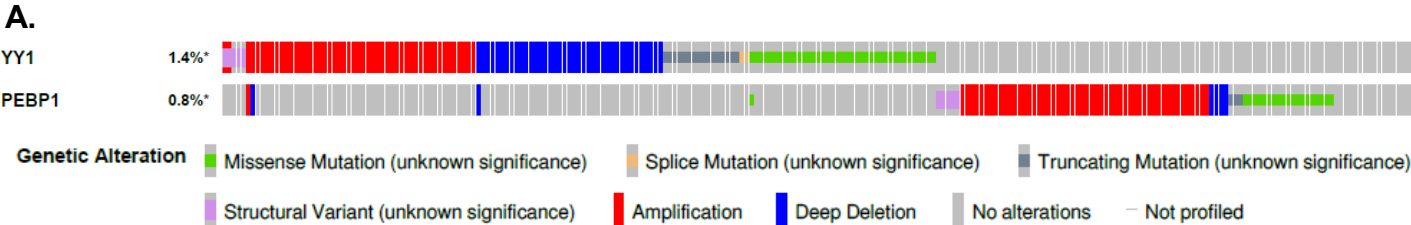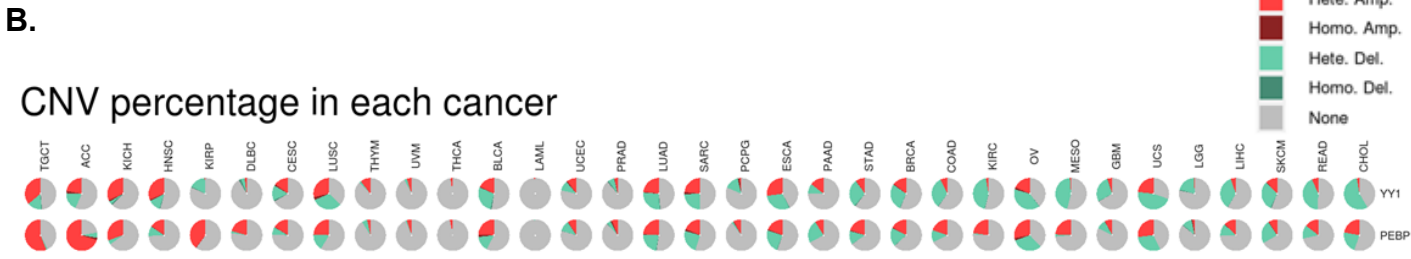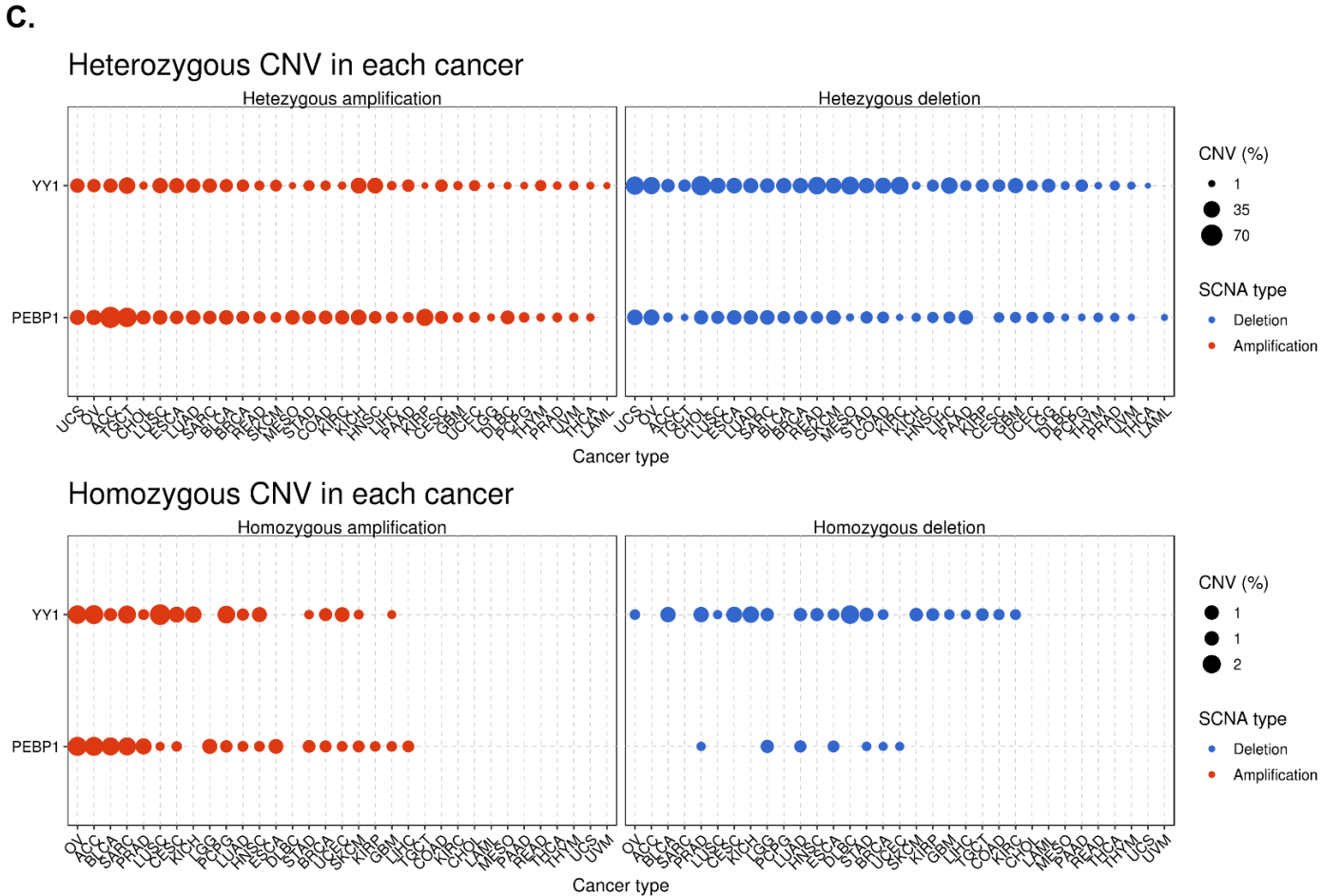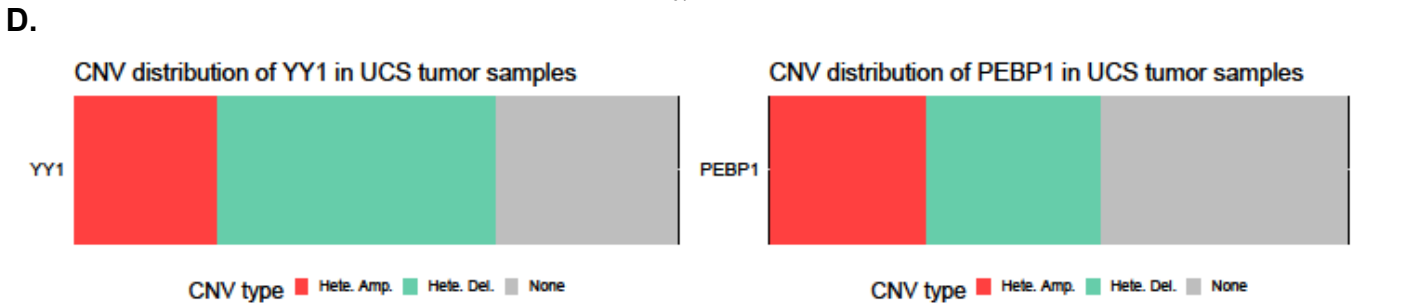

Figure S4

Supplement: Supplementary file 1 [file cancers-15-04932-s001.zip › Figure S4.pdf]
